# Insights Into Insect Vector Transmission and Epidemiology of Plant-Infecting Fijiviruses

**DOI:** 10.3389/fmicb.2021.628262

**Published:** 2021-02-24

**Authors:** Lu Zhang, Nan Wu, Yingdang Ren, Xifeng Wang

**Affiliations:** ^1^State Key Laboratory for Biology of Plant Diseases and Insect Pests, Institute of Plant Protection, Chinese Academy of Agricultural Sciences, Beijing, China; ^2^Institute of Plant Protection, Henan Academy of Agricultural Sciences, Zhengzhou, China

**Keywords:** plant-infecting fijiviruses, planthopper vectors, transmission, molecular determinants, virus epidemics

## Abstract

Viruses in genus *Fijivirus* (family *Reoviridae*) have caused serious damage to rice, maize and sugarcane in American, Asian, European and Oceanian countries, where seven plant-infecting and two insect-specific viruses have been reported. Because the planthopper vectors are the only means of virus spread in nature, their migration and efficient transmission of these viruses among different crops or gramineous weeds in a persistent propagative manner are obligatory for virus epidemics. Understanding the mechanisms of virus transmission by these insect vectors is thus key for managing the spread of virus. This review describes current understandings of main fijiviruses and their insect vectors, transmission characteristics, effects of viruses on the behavior and physiology of vector insects, molecular transmission mechanisms. The relationships among transmission, virus epidemics and management are also discussed. To better understand fijivirus-plant disease system, research needs to focus on the complex interactions among the virus, insect vector, insect microbes, and plants.

## Introduction

Plant-infecting fijiviruses have double-shelled, icosahedral particles approximately 70 nm in diameter, with spherical, short surface spikes (A spikes) on each of the 12 vertices of the icosahedron (Harding et al., 2006; Attoui et al., 2012). The outer shell is very fragile, leaving the inner shell with 12 B spikes (Teakle and Steindl, 1969; Hatta and Francki, 1977). The viral genome, which contains 10 segmented double-stranded RNAs (dsRNA) varying from approximately 1.8–4.5 kb, is approximately 29 kb within the core capsid of the virus particle (Milne et al., 1973). Segments 1–4, 6, 8, and 10 are monocistronic (only one open reading frame, ORF), while segments 5 (some fijiviruses), each of 7 and 9 of plant-infecting fijiviruses contains two ORFs (Azuhata et al., 1992; Isogai et al., 1998; Firth and Atkins, 2009; Shimizu et al., 2011; Huang and Li, 2020).

Fijiviruses are transmitted by delphacid planthoppers in a persistent-propagative manner (Pu et al., 2012). In general, after eggs hatched, the nymphs will develop to adults through five instars and both nymphs and adults can transmit viruses (Holder and Wilson, 1992). Planthoppers acquire fijiviruses when feeding in infected plants using their piercing-sucking mouthparts. Then, the virions enter and replicate into the midgut epithelia. After replication, the newly assembled virions disseminate into the hemolymph or other tissues followed by moving into the salivary glands. Finally, the virions are released from saliva to plant phloem cells when feeding (Hogenhout et al., 2008; Danthi et al., 2010). The viruses replicate and induce small tumors or enations in phloem cells of susceptible plants in families Cyperaceae, Gramineae, and Liliaceae (Huang and Li, 2020).

In nature, plant infecting fijiviruses are only spread by planthoppers vectors except garlic dwarf virus (GDV) through vegetative propagation materials (Nault, 1994). In most cases, fijivirus epidemics around the world can largely be attributed to the population density and transmission efficiency of their planthopper vectors among the different crops or gramineous weeds. Understanding the transmission mechanisms is crucial for accurate forecast and management. This review summarizes current insights of vectors for different fijiviruses, effect of viruses on the behavior and physiology of vectors, molecular determinants involved in the interaction between vector insects and the viruses, and ecological impacts of transmission biology on disease epidemiology.

## Fijiviruses, Plant Hosts, Disease Description and Distribution

Eight plant-infecting fijiviruses (Table 1) and one insect-specific fijivirus, Nilaparvata lugens reovirus, have been acknowledged by The International Committee on Taxonomy of Viruses, ICTV) (Attoui et al., 2012). Recently, another insect specific fijivirus, Psammotettix alienus reovirus, was also reported (Fu et al., 2020). Common plant hosts include flatsedge (*Juncellus serotinus* [Rottb.] C. B. Clarke) and variable flatsedge (*Cyperus difformis* L.) in family *Cyperaceae*, oat (*Avena sativa* L.), rice (*Oryza sativa* L.), sugarcane (*Saccharum officinarum* L.), maize (*Zea mays* L.), barley (*Hordeum vulgare* L.), rye (*Secale cereale* L.), wheat (*Triticum aestivum* L.), and pangola grass (a sterile triploid of *Digitaria decumbens* Stent) in family Gramineae, and garlic (*Allium sativum* L.) in family Liliaceae (Boito and Ornaghi, 2008; Li et al., 2012; Lefkowitz et al., 2018; Wu et al., 2020).

**TABLE 1:** Species of plant-infecting fijiviruses, their insect vectors and transmission characteristics.

**Species**	**Vector**	**Acquisition**	**Latency period**	**Inoculation**	**Transovarial transmission**	**Citations**
**Subgroup 1**						
Fiji disease virus	*Perkinsiella saccharicida* Kirkaldy, *P. vituensis*, *Perkinsiella vastatrix* Breddin	2 h	12–14 days	1 day	Yes (no exact data for efficiency)	Mungomery and Bell, 1933; Toohey and Nielsen, 1972; Chang, 1977; Egan and Ryan, 1986; Hughes et al., 2008
**Subgroup 2**						
Mal de Río Cuarto virus	*Delphacodes kuscheli* Fennah, *Chionomus haywardi* Muir, *Peregrinus maidis* Ashmead, *Toya propinqua* Fieber, *Caenodelphax teapae* Fowler, *Pyrophagus tigrinus* Remes Lenicov, *Tagosodes orizicolus* Muir	5 h	10 days	30 min	Not assessed	Remes Lenicov et al., 1985; Velázquez et al., 2003, 2006, 2017; Mattio et al., 2008
Maize rough dwarf virus	*Laodelphax striatellus* Fallén	10–15 min	10–15 days	15–30 min	Yes, max. 4%	Harpaz and Klein, 1969; Conti, 1974; Achon et al., 2013
Pangola stunt virus	*Sogatella furcifera* Horváth, *Sogatella kolophon* Kirkaldy	2 days	15–21 days	2–4 days	No	Greber et al., 1988; Teakle et al., 1988
Rice black streaked dwarf virus	*Laodelphax striatellus* Fallén, *Unkanodes sapporona* Matsumura, *U. albifasci*a Matsumura	30 min	7 days	5 min	No	Shinkai, 1962; Shikata and Kitagawa, 1977; Nault, 1994; Wu et al., 2020
Southern rice black streaked dwarf virus	*Sogatella furcifera* Horváth	5 min	2–6 days	30 min	No	Jia et al., 2012; Pu et al., 2012; Zhou et al., 2013
**Subgroup 3**						
Oat sterile dwarf virus	*Javesella pellucida* Fabricius, *Calligypona pellucida* Fabr.	30–60 min	3–4 weeks	30 min	Yes, 0.2%	Vacke, 1966; Milne and Lovisolo, 1977
**Subgroup 4**						
Garlic dwarf virus	Unknown					

**FIGURE 1:**
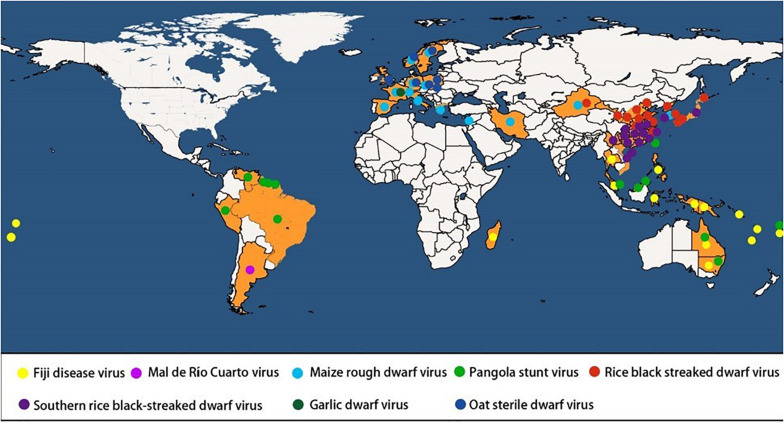
Distribution of plant-infecting Fijiviruses in the world.

Fiji disease virus (FDV), the first of eight known plant infecting fijiviruses, was reported in 1886 in Fiji (Egan et al., 1989). By 1906, the virus had destroyed thousands of acres of sugarcane (Hughes and Robinson, 1961). Now, FDV is known to cause this serious disease of sugarcane in Southeast Asian and Pacific countries, including New Guinea, Fiji, Australia, Madagascar, Vanuatu, the Philippines, and Samoa (Harding et al., 2006; Magarey et al., 2019). Its only known naturally infected host is sugarcane (*S. officinarum* L.), but other *Saccharum* species, *Sorghum* species, and maize can be experimentally inoculated with viruliferous planthoppers (Hughes and Robinson, 1961; Dhileepan et al., 2003). GDV, the only fijivirus that infects *Allium* species, has only been reported from southeastern France where it has caused occasional epidemics since 1988 (Lot et al., 1994). Although it is considered to be of low economic importance because of its limited distribution, the epidemics have caused high yield losses (Lot et al., 1994). Maize rough dwarf virus (MRDV) was initially found in northern Italy and then several European countries, where it had severe outbreaks because of the planting of maize hybrids that have higher yields but are more susceptible to the virus (Milne and Lovisolo, 1977; Marzachi et al., 1996). The hosts on which MRDV has so far been found naturally occurring are maize and some gramineous weeds, including *Digitaria sanguinalis* (L.) Scopoli, *Echinochloa crus-galli* (L.) P. Beauv, and *Cynodon dactylon* (L.) Persoon (Lovisolo, 1971). Mal de Río Cuarto virus (MRCV), initially reported as a strain of MRDV, was first reported at the end of the 1960s in maize fields in Río Cuarto County in Argentina and is now a major constraint to maize production in Argentina (Nome, 1981; Distefano et al., 2002). In addition to maize, it can also infect winter small grains (barley, oat, rye, and wheat), spring–summer grains (millet and sorghum) as well as several annual and perennial weeds (Nome, 1981; Pardina et al., 1998; Ornaghi et al., 2000). Oat sterile dwarf virus (OSDV), originally studied simultaneously in the former Czechoslovakia and in Sweden (Catherall, 1970), causes dwarfing, a dark blue-green leaf color, and profuse tillering in infected oat plants, which remain green and grass-like at harvest but lack heads. The same virus may also have been found in Netherlands, Finland, Poland, and Britain (Milne and Lovisolo, 1977). Pangola stunt virus (PaSV), first described as a devastating disease of pangola grass in Surinam (Dirven and van Hoof, 1960), is a serious threat to the cultivation of pangola grass, a sterile triploid of *Digitaria decumbens* Stent grown as a pasture grass on millions of hectares throughout the world, particularly in Florida, the Caribbean islands, and Central and South America. The virus has now been reported in other countries (regions), including Guyana, Brazil, Peru, Fiji, and Taiwan Province of China (Karan et al., 1994). Rice black streaked dwarf virus (RBSDV) was first found in Japan, where it has been present for decades but was only recognized as distinct from rice dwarf virus by Kuribayashi and Shinkai (1952). Now, it is considered to be the causal agent of rice black streaked dwarf and maize rough dwarf diseases, responsible for intermittent epidemics in East Asia and substantial yield losses over the last decades. Rice, maize, wheat, oats, and barley are its natural hosts with the similar symptoms as those of MRDV (Azuhata et al., 1993; Wu et al., 2020). In 2008, a RBSDV-like new virus, southern rice black streaked dwarf virus (SRBSDV), was reported in the south of China (Zhou et al., 2008) and caused serious yield losses in China, Vietnam and Japan during 2010s (Zhou et al., 2013). The global distribution of plant-infecting fijiviruses is shown in Figure 1.

## Vector Insects

Plant-infecting fijiviruses are primarily transmitted by delphacid planthoppers, which belong to a large, diverse superfamily Fulgoroidea, even though the insect vector of GDV has not been found yet. The species (*Fiji disease virus*, *Mal de Río Cuarto virus*, *Maize rough dwarf virus*, *Pangola stunt virus*, *Rice black streaked dwarf virus*, *Southern rice black streaked dwarf virus*, *Oat sterile dwarf virus* and *Garlic dwarf virus*) in genus *Fijivirus* and any known vectors are described in Table 1. FDV is transmitted by *Perkinsiella saccharicida* Kirkaldy, *Perkinsiella vituensis*, and *Perkinsiella vastatrix* (Toohey and Nielsen, 1972; Mungomery and Bell, 1933; Hughes et al., 2008). Both MRDV and RBSDV are naturally transmitted by the small brown planthopper *Laodelphax striatellus* Fallén (Achon et al., 2013; Nault, 1994). Two other planthoppers *Unkanodes sapporona* Matsumura and *Unkanodes albifascia* are not of great importance for RBSDV epidemic because of their low density in the field, though they also transmit RBSDV (Wu et al., 2020). Different planthopper species in Argentina can transmit MRCV, but *Delphacodes kuscheli* Fennah is the most plentiful and recurring species in areas where the virus is endemic (Remes Lenicov et al., 1985). Many other planthopper species, including *Chionomus haywardi* Muir, *Peregrinus maidis* Ashmead, *Toya propinqua* Fieber, *Caenodelphax teapae* Fowler, *Pyrophagus tigrinus* Remes Lenicov and *Tagosodes orizicolus* Muir are also known to transmit MRCV (Velázquez et al., 2003, 2006, 2017; Mattio et al., 2008). MRCV epidemics are largely related to the abundance, frequency and transmission efficiency of the vectors in early stages of the maize crop (Ornaghi et al., 1999; Laguna et al., 2002; Velázquez et al., 2003). The planthopper *Javesella pellucida* Fabricius is the natural vector of OSDV, but the leafhopper *Calligypona pellucida* Fabr also transmits OSDV (Milne and Lovisolo, 1977). *Sogatella kolophon* Kirkaldy and *Sogatella furcifera* were shown to vector PaSV (Greber et al., 1988; Teakle et al., 1988). SRBSDV has been identified as being transmitted by *S. furcifera*, but *L. striatellus* can also acquire it but not transmit to plants (Jia et al., 2012; Pu et al., 2012; Zhou et al., 2013).

## Transmission Characteristics

Like other persistent and propagative viruses, plant-infecting fijiviruses must initially infect and replicate in the epithelial cells of the insect’s midgut, and then disseminate in different tissues, eventually spread to salivary glands, where are excreted to plants through saliva during insect feeding (Hogenhout et al., 2008; Mainou and Dermody, 2012). To complete the whole cycle of virus transmission, vector insects must undergo specific periods for acquisition, latency and inoculation. Some fijiviruses can be transovarially transmitted with low efficiency.

### Acquisition

The acquisition periods range from several minutes to a few days for most plant-infecting fijiviruses, which have been mainly found in the phloem (Francki and Boccardo, 1983; Conti, 1984). Generally, the virus is more efficiently acquired by nymphs than by adults. In two similar examples, FDV is transmitted by *P. saccharicida* only if the virus is acquired by first-instar nymphs, but not older instars or adults, and first-instar nymphs of *D. kuscheli* transmit MRCV with higher efficiency compared with older instar nymphs (Hutchinson and Francki, 1973; Arneodo et al., 2005).

### Latency and Inoculation

Once inside the planthoppers, the virus multiplies during a 1–4 weeks latency period when it is also disseminating into other tissues, including the salivary glands (Greber et al., 1988). When the latency period is complete, that is, the viral titre has reached a certain level, the insects can frequently transmit virus in their lifetime (Conti, 1984; Hughes et al., 2008; Argüello Caro et al., 2013; Hajano et al., 2015). For RBSDV, the minimum inoculation threshold has been shown to be as short as 5 min (Shikata and Kitagawa, 1977), but the minimum so far observed for MRDV is 15–30 min (Conti, 1974).

### Transovarial Transmission

Only a few studies have shown that fijiviruses are transovarially transmitted in their planthopper vectors (Chang, 1977; Conti, 1985). For FDV, transovarial transmission in *P. saccharicida* was reported but no data were included (Chang, 1977). Transovarial transmission of MRDV in *L. striatellus* was reported to be about 4% (Harpaz and Klein, 1969), but none of 300 progenies from *L. striatellus* eggs deposited on sorghum were viruliferous (Harpaz, 1972). For OSDV, only 0.2% transovarial transmission was found in one case (Vacke, 1966) and no evidence found in another case for *J. pellucida* (Lindsten, 1974). Also, RBSDV and SRBSDV are not transmitted through the eggs of the vector *L. striatellus* and *S. furcifera* (Shinkai, 1962; Pu et al., 2012). Times for acquisition, latency and inoculation vary among the different combinations of viruses and vectors and are shown in Table 1.

## Virus Effects on Behavior and Physiology of Vector Insects

Because fijiviruses must infect, multiply, and spread in their vector insect cells, virus infections have multiple effects on the behavior and physiology of their vector insects. Reports on these effects, however, are contradictory. Adverse effects include extending the nymphal stages, shortening the lifespan of adults, decreasing survival rate or fecundity (Harpaz and Klein, 1969; Nakasuji and Kiritani, 1970; Wei and Li, 2016). For example, Harpaz and Klein (1969) reported that MRDV viruliferous females of *L. striatellus* laid 30–50% fewer eggs than non-viruliferous females did and that the viability of these eggs was poor (14% vs. 99% for hatch from non-viruliferous). In those cases where hatching did occur, the incubation period was longer by up to 3 days (about 25%) than that of eggs from non-viruliferous females, and the mortality of the resulting larvae was high. Similarly, compared with non-viruliferous *S. furcifera*, SRBSDV-viruliferous females deposited fewer eggs and the viruliferous nymphs spent longer time developing into adults (Xu et al., 2014). In contrast, Zhang et al. (2014) reported that SRBSDV infection in rice led to an increase in the fecundity of *S. furcifera* and population size of macropterous adults (Zhang et al., 2014).

Feeding behavior of SRBSDV-viruliferous *S. furcifera* also differs from that of non-viruliferous insects; the frequency of phloem sap ingestion of viruliferous *S. furcifera* is significantly higher, but total feeding duration does not increase markedly (Xu et al., 2014). When SRBSDV-viruliferous *S. furcifera* feed on uninfected plants, they spend longer time in salivation and have more frequent phloem sap ingestion than did non-viruliferous insects (Xu et al., 2014). These behavioral alterations might be adapted to the benefit of virus acquisition and inoculation (Lei et al., 2016). The infection of certain plants by MRDV can also modify the capacity of those plants to support MRDV vectors (Klein and Harpaz, 1969; Harpaz, 1972). For example, *L. striatellus* is unable to survive on non-infected *Cynodon dactylon* (L.) Pers. (Bermuda grass) for longer than 4–7 days and does not molt or lay eggs on this plant, but it can survive and breed successfully when the plants are infected with MRDV or the planthopper is already MRDV-viruliferous when placed on the plant (Klein and Harpaz, 1969).

These adverse or beneficial changes might be due to the changes of physiological and metabolic components caused by virus-plant/insect interactions. The physiology is altered in MRCV-infected wheat plants; the contents of total soluble sugar, starch, protein and malondialdehyde levels increase noticeably, but chlorophyll content decreases considerably. These variations are indicative of oxidative damage associated with biotic stress in these plants (Di Feo et al., 2010). MRCV-infected wheat plants had more than 3,000 differentially accumulated transcripts (DATs) at 21 days post inoculation, and exhibited higher levels of soluble sugars, starch, trehalose 6-phosphate (Tre6P), and organic and amino acids, but decreased transcripts levels for TaSWEET13, which are involved in sucrose phloem loading (de Haro et al., 2019). Similarly, the physiology and metabolism of viruliferous insect vectors change greatly. In SRBSDV-viruliferous *S. furcifera* with viral titer-specific monotonic transcriptome changes: 1,906 genes increase and 1,467 genes decrease in expression (Wang et al., 2016). In gas chromatography-time of flight-mass spectra, the major categories of metabolites differentially regulated after SRBSDV infection are nucleic acids and fatty acids, whereas the compounds relative to tricarboxylic acid cycle, sugars, and polyols are differentially regulated after temperature stress (Zhang et al., 2018).

## Competition or Synergism of Co-Infecting Viruses in Relation to Transmission

Mal de Río Cuarto virus has been detected in co-infection with an isolate of maize yellow striate virus (MYSV, genus *Cytorhabdovirus*, family *Rhabdoviridae*) in maize (Dumón et al., 2015, 2017). Both viruses can naturally infect maize and several grasses through transmission by *D. kuscheli*. Although most of planthoppers could be viruliferous after feeding on mixed-infected plants, planthoppers with notably higher MRCV titers are able to transmit the virus, meaning that efficient MRCV transmission is positively correlated with virus accumulation in the insect (Argüello Caro et al., 2013). Plants doubly-infected by MRCV and the rhabdovirus showed typical symptoms of MRCV earlier than that single infected with MRCV, but the planthoppers fed on doubly-infected plants only acquired lower MRCV titers and transmitted inefficiency, indicating that these two viruses have antagonism in host plants and vector insects (Dumón et al., 2017).

Conversely, the epidemics of rice ragged stunt virus (RRSV, genus *Oryzavirus*, family *Reoviridae*), transmitted by *Nilaparvata lugens* Stal, has become more frequent in southern China since its co-infection with SRBSDV during 2010s (Wang et al., 2013). Rice plants doubly-infected by both viruses showed earlier and enhanced symptoms. *S. furcifera* and *N. lugens*, respectively, acquired SRBSDV and RRSV from doubly-infected plants with higher efficiency (Li et al., 2014). Furthermore, the non-viruliferous *N. lugens* significantly preferred feeding on virus-free plants, whereas viruliferous *N. lugens* preferred SRBSDV-infected rice plants (Wang et al., 2013). The attractiveness of the SRBSDV- or RRSV-infected rice plants to planthoppers is mainly caused by the changes of rice volatiles (Lu et al., 2016). Fecundity of *N. lugens* feeding on SRBSDV-infected rice plants is higher than those that fed on uninfected plants, but nymphal duration of males is significantly prolonged (Xu et al., 2016). Thus, these two viruses may alter the vectors’ host preference to the benefit of their spread.

## Insect Microbes

Various microbes in vector insects have also been known to directly affect virus infection or transmission (Mousson et al., 2012; Kliot and Ghanim, 2013; Jupatanakul et al., 2014). The symbiont *Chromobacterium* could reduce the susceptibility *of Aedes aegypti* to dengue virus infection and another symbiont *Wolbachia* in *Aedes albopictus* decreased virus transmission efficiency through decreasing the titer of viruses in host cells (Moreira et al., 2009; Jupatanakul et al., 2014; Kliot et al., 2014). On the contrary, acquisition and transmission of tomato yellow leaf curl virus by *Bemisia tabaci* were significantly enhanced by *Rickettsia* (Kliot et al., 2014). Moreover, the microbiota might contribute to the higher fecundity of *L. striatellus*, which in turn may be associated with the outbreaks of the virus (Liu et al., 2020). Besides microbes in vector insects, some insect-specific viruses have also been found in *L. striatellus* or *S. furcifera* (Wu et al., 2018, 2019). These new insect-specific viruses might affect fijivirus replication and transmission.

## Molecular Mechanisms Involved in Transmission

Replication and spread of plant-infecting fijiviruses in different organs of their vector insects require specific interactions between virus and vector components. Some of these viral determinants and insect components which might be related to transmission of plant-infecting fijiviruses by their respective insect vectors have recently been identified.

### Viral Determinants Involved in Viral Transmission

Complete genomic sequences are available for FDV, MRCV, MRDV, RBSDV, and SRBSDV, but only partial sequence genome information is available for OSDV. For RBSDV and SRBSDV, the whole genome encodes 13 viral proteins, including six putative structural proteins: P1 (the RNA-dependent RNA polymerase), P2 (major core structural protein), P3 (capping enzyme), P4 (outer shell B-spike protein), P8 (minor core protein), and P10 (major outer capsid protein), seven putative non-structural proteins P5-1, P5-2, P6, P7-1, P7-2, P9-1, and P9-2 (Attoui et al., 2012). These viral proteins can directly or indirectly affect viral transmission. The major outer capsid protein P10 plays key roles for virus invasion and transmission in vector insects (Than et al., 2016). P7-1 forms the tubule structures that serve as vehicles to transport the virions across the basal lamina and enable intercellular movement of the virus within insect cells (Jia et al., 2014, 2016).Non-structural proteins P9-1, P6 and P5-1 indirectly contribute to virus transmission since they constitute cytoplasmic inclusion bodies called viroplasms where virus replication takes place and in turn high replication and consequent high virus titers result in successful transmission (Zhang et al., 2008; Maroniche et al., 2010; Jia et al., 2012).

In plants and insects, small interfering RNA (siRNA) pathway plays a key role in antiviral defense (Ding, 2010; Zvereva and Pooggin, 2012; Gammon and Mello, 2015). In vector insects, this pathway may affect virus titers, persistence and transmission efficiency. Virus-derived siRNAs (vsiRNAs) mainly given rise by Dicer-2 in *Drosophila melanogaster* and mosquitoes to limit viral infection have been extensively studied (Fragkoudis et al., 2009; Blair, 2011; Donald et al., 2012). VsiRNAs accumulate in RBSDV-viruliferous *L. striatellus*, indicating that fijivirus replication might induce an RNAi-mediated antiviral response in its insect vector (Li et al., 2013). Similarly, SRBSDV infection induced the siRNA pathway in the midguts of an incompetent vector *L. striatellus*. Interfering with Dicer-2 significantly increased virus replication in midgut epithelial cells of *L. striatellus*, so that viral titers reached a threshold and disseminated to the *L. striatellus* midgut muscle layer (Lan et al., 2016). de Haro et al. (2017) also provided evidence that the silencing mechanism of plants and insect vectors for MRCV could distinguish viral genomes, and thus produced different vsiRNAs, which suggested fijiviruses might encounter different and distinctive defense strategies both in host plants and vector insects.

### Insect Components Involved in Transmission

**FIGURE 2:**
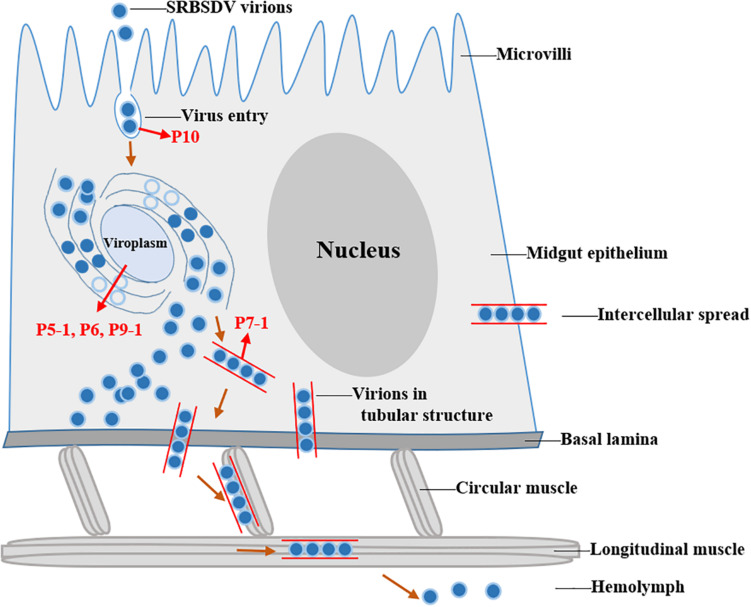
Model of SRBSDV replication and transmission in midgut epithelium of *Sogatella furcifera*. SRBSDV enters into midgut epithelium by endocytosis through the interactions between the major outer capsid protein P10 and receptors in cell membrane and replicates in viroplasms, which are composed of non-structural proteins P9-1, P6, and P5-1. After replication, virions-containing P7-1 tubular structures directly pass across the basal lamina, traffic along the internal muscle tissues and then disseminate virions into hemolymph.

For successful transmission, fijiviruses have to break through various transmission barriers in midgut, salivary gland, and defense immune response of their vector insects. Multiple interactions among virus and vector components are necessary for finishing above processes (Liu et al., 2018). Based on a yeast two-hybrid system (Y2H), proteins in vector *S. furcifera* were identified to interact with P6, P7-1 or P10 of SRBSDV. Five proteins (bromodomain-containing protein [BRD], succinyl-CoA ligase [ADP-forming] subunit beta [SUCLSB], 40S ribosomal protein SA [RPSA], toll-interacting protein [TOLIP] and signal transducing adapter molecule 1 [STAM1]) interacted with SRBSDV P6, and they are mainly involved in gene transcription, protein translation, protein post-translational modification and protein synthesis (Zhao et al., 2019). In *S. furcifera*, 18 proteins were confirmed interacting with SRBSDV P7-1 and six of them (neuroglian, myosin light chain 2 [MLC2], polyubiquitin, E3 ubiquitin ligase, ribophorin II, and profilin) exhibited different levels in five organs: neuroglian, MLC2, polyubiquitin and profilin highest in the gut, but ribophorin II and E3 ubiquitin ligase highest in the salivary glands and hemolymph, respectively, which indicated that they might play specific roles in viral progresses in different organs (Mar et al., 2014). Besides, 28 proteins interacted with the P10 of SRBSDV were identified. The mRNA level of vesicle-associated membrane protein 7 (VAMP7) was highest in the gut, but vesicle transport V-SNARE protein (Vti1A) and Growth hormone-inducible transmembrane protein (Ghitm) was highest in malpighian tubule compared with other tissues (Than et al., 2016). Based on the known data, we proposed a model for SRBSDV trafficking in midgut epitheliums of its insect vector, *S. furcifera* (Figure 2). SRBSDV virions invade into midgut epithelial cells by endocytosis through the interactions between the major outer capsid protein P10 and receptors in insects. Then the virus replicates in the viroplasm where P5-1, P6, P9-1 play vital roles in viroplasm formation. Insect proteins interacting with P6 might also assist recruit P5-1 and P9-1. After replication, the virions disseminate into hemolymph freely or in tubular vehicles through the interaction between P7-1 and cytoskeleton.

## Virus Epidemics and Management

As discussed earlier, transmission by insect vectors is essential for the spread and epidemics of plant-infecting fijiviruses because they are not transmitted by seeds or mechanical means. Here, we discuss the impacts of transmission on virus epidemics in different crops.

### Relationship Between Transmission and Epidemics

Fiji disease virus can cause Fiji leaf gall (FLG), a major problem for Australian sugarcane industry. The virus epidemics led to serious yield losses during 1970–1990 because FLG-susceptible cultivar NCo310 was planted in a large area and the vector *P. saccharicida* was present at a high density (Ryan, 1988). Besides, the vector planthopper of FDV occurs in all sugarcane-growing areas of Queensland and New South Wales. If only a species of *Perkinsiella* in the sugarcane on the east coast of Australia, the movement of insects from areas without FDV to areas with FDV is not important (Egan and Ryan, 1986; Egan et al., 1989; Dhileepan and Croft, 2003). On the other hand, if a species exists in northern Queensland but not in southern Queensland, and transmits FDV more efficiently than the current population of *P. saccharicida* in southern Queensland, then the migration of these northern insects will pose a risk to the southern region (Magarey et al., 2019).

**FIGURE 3:**
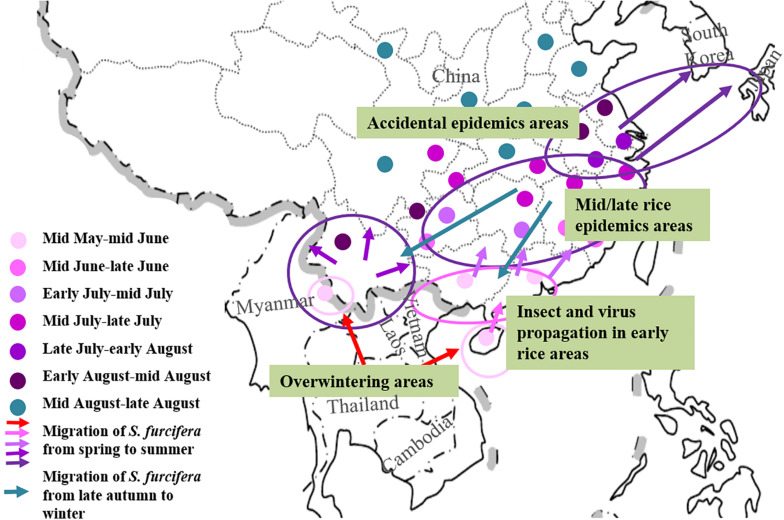
The relationship between long distance migration of *S. furcifera* and SRBSDV epidemic in Eastern and Southeastern Asia.

Mal de Río Cuarto disease is the most important maize viral disease in Argentina (March et al., 1993, 2002; Lenardón et al., 1998). Although the vector *D. kuscheli* does not reproduce on maize, it breeds on MRCV susceptible winter cereals such as oat, wheat and rye, which serve as virus reservoirs (Trumper et al., 1996). Generally, viruliferous *D. kuscheli* migrate from senescent winter cereals to maize and then transmit MRCV when it feeds on maize (Ornaghi et al., 1993, 1999; March et al., 1995). If the major immigration period of high density of insect vectors is consistent with maize early growth stages (the first 3 weeks after maize emergence) of susceptible maize genotypes, severe outbreaks will occur (Ornaghi et al., 1993). Similarly, the epidemics of maize rough dwarf disease caused by MRDV in Spain has close relationship with the population density and distribution of its vector *L. striatellus*, especially at early stages of crop development, and to the susceptibility of the maize varieties (Conti, 1976; Trumper et al., 1996). In northern China, a double-cropping system for interplanted and intercropped maize and wheat was widely implemented in the 1980s. Such cropping system provide enough food sources for vector *L. striatellus*, resulting in its high population density and the opportunity for overwintering. In addition, the virus can be transmitted easily among different hosts, leading to outbreaks of maize rough dwarf diseases in these regions (Ren et al., 2016). As already mentioned, two fijiviruses, RBSDV and SRBSDV, are known to infect rice and cause serious yield losses in East Asian countries. For RBSDV or SRBSDV epidemics, migration and virus transmission of *L. striatellus* or *S. furcifera* among the different crops or gramineous weeds are essential. Field investigations have shown that outbreaks of SRBSDV-induced rice black streaked dwarf disease usually coincide with mass long distance migration of *S. furcifera* between or within Vietnam, Myanmar, Japan, South Korea, and China (Figure 3). Typhoons may carry migrating insects from southern China to northern Vietnam in the late fall or early winter, and then overwinter in tropic regions (Zhao et al., 2011; Matsukura, 2013), revealing that vector *S. furcifera* plays a crucial role in the SRBSDV epidemics. Under normal circumstances, the viruliferous macropterous adults are driven by winds to the Pearl River Basin (Guangdong) and Honghe Prefecture (Yunnan) in March. In central and eastern China, initial infection usually occurs in late spring and early summer from the viruliferous *S. furcifera* population that migrates with air flows from southern provinces of China and northern Vietnam or Myanmar (Zhou et al., 2013). In most regions with RBSDV epidemics in China, the disease incidence is closely related to the population density and viruliferous rate of the vector *L. striatellus*, which can finish its life cycle and seasonally migrate in wheat, barley, and some gramineous weeds in most regions (Wu et al., 2020).

### Management

Integrated management strategies are urgently needed to reduce heavy losses caused by fijiviruses. At present, an economical and effective method for managing diseases is controlling vector insects on account of lacking disease-resistant varieties. Covering plant seedling nurseries with insect-proof nets is broadly used to control black streaked dwarf diseases caused by RBSDV or SRBSDV in China. Postponing sowing time of maize is available to prevent the peak period of *L. striatellus* planthopper immigration coinciding with the susceptible period of the rice. Seed dressing with pesticides is also recommended before sowing, and then the seedlings are applied pesticides three to 5 days before transplanting (Ren et al., 2016). For the management of Mal de Río Cuarto disease, the most important management practice is to advance the sowing so that the vector population peaks do not coincide with the emergency of the maize plants consulting a predictive model based on temperature variables and the disease intensity (March et al., 1995; Satapathy, 1998). Except managing insects, using virus-free vegetative materials and resistant varieties, removing infected stools or crops, and quarantining are existing effective methods to control FLG in Australia (Egan and Ryan, 1986; Dhileepan and Croft, 2003).

## Concluding Remarks and Prospects

In general, field epidemics of plant fijiviruses have three typical stages: serious outbreak for 2–3 years, gradual mitigation over the next 2–3 years, and negligible disease for several years; thus, the disease tends to be intermittent over a longer period (Achon et al., 2013; Wu et al., 2020). Although existing virus management practices have played an important role during virus management, the underlying reasons for the epidemics are still poorly understood. Fijivirus epidemics are related to various biotic and non-biotic factors, so to better understand this complex pathosystem, and then provide accurate predictions and control measures, further study of transmission biology should be explored to reveal the reasons of the intermittent epidemics. Many aspects need to be addressed, including (a) the identification of determinants for vector specificity and viruses breaking through various transmission barriers in their vector insects. The roles of viral proteins, vector proteins, and insect symbionts in determining vector specialization also need to be further elucidated, which can provide targets for molecular design to inhibit transmission (Hajano et al., 2020). (b) Epigenetic RNA or DNA methylation of viral or host genes and changes in gene splicing upon virus infection can also affect interactions between the virus and vector insect and change vector specificity and transmission efficiency (Rivera-Serrano et al., 2017; Usama et al., 2019). (c) Global climate warming, organic farming methods to reduce the use of pesticides, and the commercialization of genetically modified crops that are resistant to lepidopterans or beetles may indirectly lead to the changes of planthopper population and the outbreaks of fijiviruses. Similar works have been done for other virus–vector insect systems (Wei and Li, 2016; Qin et al., 2018; Wang et al., 2019; Liu et al., 2020), but whether these results also apply to fijiviruses and their vector insects needs to be determined.

To further advance protection against these viruses, multidisciplinary approaches are needed. (a) Cooperative research in plant pathology, insect ethology, ecology and other disciplines can help clarify mechanisms underpinning the four-way interactions in the plant–insect vector–virus–environment network. (b) Entomologists, plant virologists, crop breeders, and ecologists can coordinate and cooperate to advance the selection, cultivation and rational distribution of resistant varieties. (c) International exchange and cooperation should be strengthened to solve disease problems from a global perspective, focusing on the main places where the diseases occur, ascertain the migratory pathways of the vectors and the source of foreign insects, determine the source of the virus, and prevent outbreaks of the disease on an international scale. (d) A variety of experimental techniques for molecular biology, biochemistry, and environmental biology can be used to comprehensively study the pathogenic mechanism of viruses and their interactions with vectors and develop virus inhibitors or new technologies to block acquisition and transmission of viruses by the vectors.

## Author Contributions

XW conceived and designed the review. LZ, NW, YR, and XW wrote the manuscript. All authors contributed to the article and approved the submitted version.

## Conflict of Interest

The authors declare that the research was conducted in the absence of any commercial or financial relationships that could be construed as a potential conflict of interest.
